# FDG altered biodistribution in white adipose tissue, a rare entity: case report and review of the literature

**DOI:** 10.1186/s41824-024-00209-5

**Published:** 2024-07-15

**Authors:** William Y. Raynor, Stephen J. Sozio, Jeffrey S. Kempf

**Affiliations:** Department of Radiology, Rutgers Robert Wood Johnson Medical School, 1 Robert Wood Johnson Place, MEB #404, New Brunswick, NJ 08901 USA

**Keywords:** PET/CT, Lymphoma, Corticosteroids, Glucocorticoids, Dexamethasone, Adipogenesis

## Abstract

**Purpose:**

Altered ^18^F-fluorodeoxyglucose (FDG) biodistribution due to patient factors such as exercise and inadequate fasting are well established causes of limited diagnostic efficacy. In addition, medications such as G-CSF are known to affect uptake of FDG by bone marrow and spleen. In this study, we present a case of increased white adipose uptake in a pediatric lymphoma patient who recently received high dose dexamethasone and review the relevant literature regarding this rare and poorly understood pattern of altered FDG biodistribution.

**Methods:**

A 14-year-old male patient diagnosed with B-cell lymphoblastic lymphoma underwent FDG-PET/CT for restaging shortly after completing an induction chemotherapy regimen. Images revealed diffuse FDG uptake localizing to white adipose tissue, attributed to the 29-day course of dexamethasone which was completed two days prior. A diagnostically adequate study with relative normalization of FDG biodistribution was obtained seven days later.

**Results:**

In our review of the literature, diffuse FDG uptake by white fat is a rare occurrence and has only been reported by a few case reports and early observational studies. In addition to patients receiving corticosteroids, other cases of medication-induced adipose remodeling such as patients receiving highly active antiretroviral therapy have been documented with similar patterns of increased white adipose tissue activity.

**Conclusion:**

Corticosteroid-induced white fat uptake of FDG is a rare phenomenon that can limit diagnostic accuracy of FDG-PET/CT and necessitate repeat imaging. Current evidence suggests that a wait period of at least one week after discontinuation of corticosteroids is sufficient to allow for decreased white fat uptake and increased diagnostic accuracy.

## Introduction

With the introduction of ^18^F-fluorodeoxyglucose (FDG) in 1976, followed by the development of the integrated positron emission tomography/computed tomography (PET/CT) scanner in the early 2000s, lymphoma assessment quickly became a major application of FDG-PET/CT (Barrington, et al. 2014). Initially, FDG/PET-CT was frequently used before treatment initiation, but it was not formally included in staging due to the scarcity of data at the time (Cheson 2018). To create uniform reporting standards, the First International Workshop was organized in Deauville, France in 2009 (Meignan et al. 2009). There it was agreed that the Deauville score (DS) would be used for visual analysis of FDG-PET findings. Previous research demonstrated that the DS had high interobserver reliability with implications for prognosis (Mikhaeel et al. 2005; Gallamini et al. 2007). In 2014, the Lugano guidelines were updated to include a revised 5-point DS for use in interim and end-of-treatment assessments (Heertum et al. 2017). The DS established benchmarks for determining whether a response to treatment was adequate or inadequate, which could be adjusted based on the clinical situation.

Fasting prior to FDG administration is a well-known necessity to avoid altered tracer biodistribution. Increased plasma glucose competes with FDG at sites of uptake, resulting in decreased FDG localization to the brain and tumor cells. Inadequate fasting also increases serum insulin, resulting in extensive skeletal muscle uptake of FDG mediated by increased expression of glucose transporter-4 (Nakatani et al. 2012). This pattern of diffuse FDG uptake by skeletal muscle is well documented and has previously been termed a “muscle scan” (Turcotte, et al. 2006). In addition to the effects of glucose and insulin, treatment-induced changes in FDG uptake have also had important implications for image interpretation. One type of medication known to influence FDG uptake is granulocyte colony-stimulating factor (G-CSF), a hematopoietic cytokine involved in the growth and development of bone marrow hematopoietic progenitor cells, which is used to manage chemotherapy-induced neutropenia in cancer patients (Hanaoka et al. 2011). When G-CSF is administered to patients undergoing chemotherapy before an FDG-PET/CT, there is a significant increase in FDG uptake in the bone marrow and to a lesser extent in the spleen, despite normally low physiological localization. This change in FDG distribution, induced by G-CSF, can resemble the progression of bone marrow disease, complicating disease staging since the bone marrow is often a target for spread of lymphoma. However, treatment with G-CSF is not the only medication-related change in FDG biodistribution that needs to be considered in patients undergoing therapy.

In this current study, a rare type of altered FDG biodistribution in a pediatric lymphoma patient, demonstrating diffusely increased uptake in subcutaneous white adipose tissue, associated with prolonged treatment of corticosteroids administered during induction chemotherapy will be presented and reviewed.

## Case presentation

**Fig. 1:**
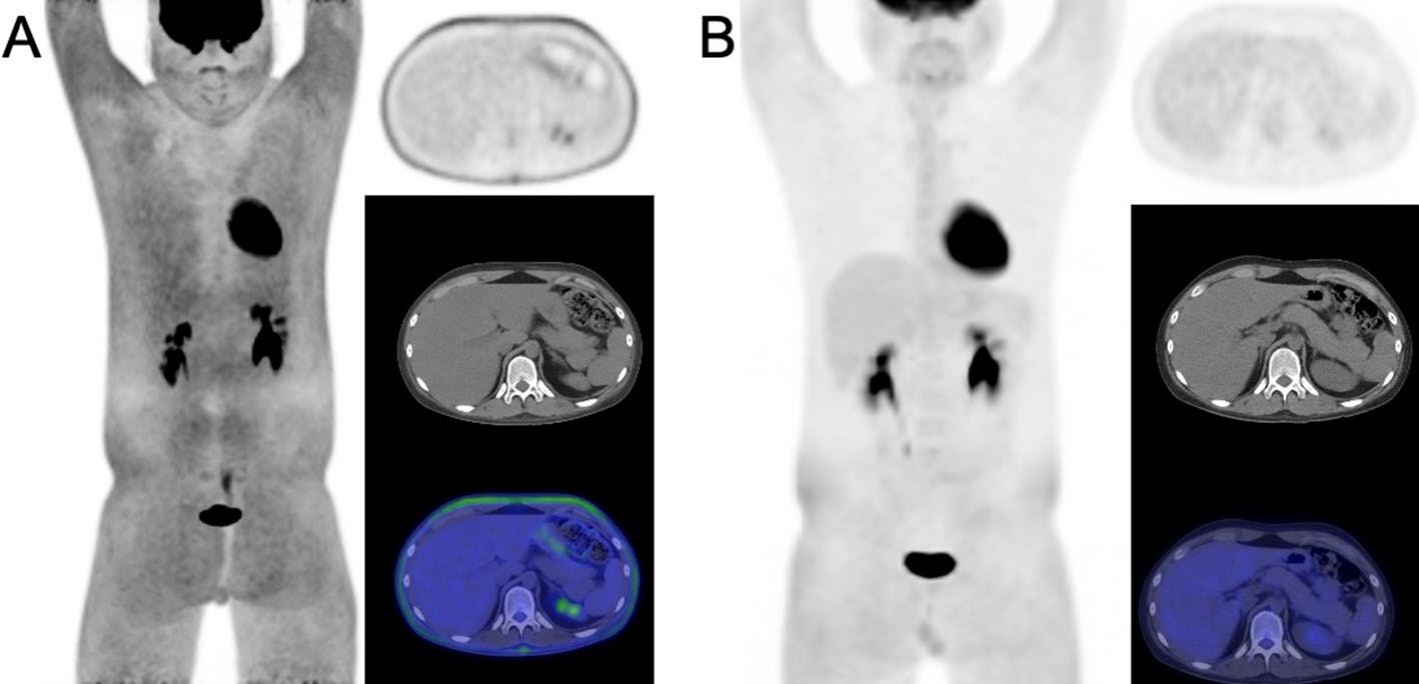
FDG-PET/CT images for restaging approximately one month after initial baseline imaging. Obtained two days after the patient finished a 29-day regimen of high-dose dexamethasone, the initial study (**A**) clearly shows diffuse FDG uptake in white adipose tissue, as seen on the maximum intensity projection (MIP), axial PET, and fused PET/CT imaging, limiting diagnostic utility. Repeat FDG-PET/CT performed 7 days later (9 days since last dose of dexamethasone) demonstrates that the increased interval since discontinuation of corticosteroids resulted in relative normalization of white fat activity in the upper extremities, chest, and abdomen, with mild persistent activity involving the white fat in the pelvis and proximal lower extremities (**B**)

A 14-year-old boy weighing 62 kg (137 lbs) underwent an FDG-PET/CT scan for the restaging of B-cell lymphoblastic lymphoma. He had fasted for 8 h prior to the scan, and his blood glucose level was recorded at 87 mg/dL when the FDG dose (7mCi) was administered intravenously. The patient’s whole body FDG-PET/CT scan showed an altered biodistribution with diffusely increased FDG uptake in the white fat tissue across the neck, chest, abdomen, pelvis, and proximal thighs (Fig. 1A). However, imaging at this time did not demonstrate any activity that could clearly be attributed to lymphoma activity in spite of the unusual pattern of tracer localization. Upon reviewing the patient’s medications, it was determined that the observed increase in white fat metabolism was due to the effects of glucocorticoids. Upon further review of the patient’s history, it was discovered that the patient had just completed a 29-day treatment with dexamethasone, taking 5 mg twice daily, which concluded two days prior to the FDG-PET/CT scan. Dexamethasone was given as part of his initial chemotherapy treatment, which also included treatment with vincristine and L-asparaginase. A follow-up scan performed a week later showed a significant reduction in FDG activity in the white fat tissue, with only slight residual uptake noted in the subcutaneous fat of the pelvis and proximal thighs (Fig. 1B). The subsequent scan was overall felt to be interpretable for treatment response, with low probability of any potential obfuscation of disease by residual activity in white adipose tissue.

## Discussion

White adipose tissue is a major hormonally active organ, releasing substrates such as adipokine, adiponectin, leptin, resistin, and tumor necrosis factor, which play pivotal roles in managing insulin sensitivity and overall metabolism (Haas et al. 2012). Structurally, white fat cells, which contain a single large lipid droplet, are involved in storing energy in subcutaneous, intramuscular, and visceral fat compartments. In comparison, brown fat cells, which contain multiple small lipid droplets in addition to a high number of mitochondria, are responsible for heat production (Wong et al. 2020). Generally, white fat is metabolically less active and demonstrates minimal physiologic FDG activity. Specifically, FDG activity in subcutaneous fat has been demonstrated to be similar across various patient populations, including healthy subjects and those with metabolic syndrome (Oliveira et al. 2015).

The phenomenon of diffuse FDG activity by white fat following corticosteroid treatment is an uncommon finding, predominantly observed in young cancer patients, with only about 20 pediatric leukemia and lymphoma cases with this phenomenon documented in a few early observational studies and case reports within the medical literature (Table 1) (Wong et al. 2020; Young et al. 2021; Kapoor et al. 2021; Sharp et al. 2012). Induction chemotherapy regimens for leukemia and lymphoma frequently incorporate corticosteroids such as prednisone or dexamethasone to be administered alongside agents such as vincristine. This combination of vincristine with corticosteroids is known to effectively decrease tumor size (Angiolillo et al. 2021). The metabolic activities of glucocorticoids, which include the breakdown and formation of lipids as well as the creation of fat cells, are believed to cause the accumulation of FDG in white fat seen in these cases (Wong et al. 2020). Specifically, the drug-induced transformation of white fat tissue into a pattern resembling Cushing's syndrome involves the differentiation of fat cells and an increase in glycolysis (Sharp et al. 2012).

**Table 1:** Human studies which describe altered biodistribution of FDG characterized by diffuse white adipose tissue activity

First Author and Affiliation (Year)	Patients, *n* (female)	Patient Population	Main Findings
Bleeker-Rovers, Radboud University Nijmegen Medical Centre, Netherlands (2004) (Bleeker-Rovers et al. 2004)	4 (0)	Adults with HIV (range: 28–56 years old), including 4 never having received HAART, 5 patients on HAART without lipodystrophy, and 4 patients on HAART with lipodystrophy	Three out of 4 HIV patients receiving HAART with clinical findings of lipodystrophy had increased FDG activity in the subcutaneous white fat. This phenomenon was not observed in patients without lipodystrophy
Sathekge, University of Pretoria, South Africa (2010) (Sathekge et al. 2010)	39 (21)	Adults and adolescents with HIV (range: 16–64 years old), including 7 drug-naive patients, 21 patients on HAART without lipodystrophy, and 11 patients on HAART with lipodystrophy	Subcutaneous white fat FDG activity was found to be significantly higher in patients on HAART with lipodystrophy compared to nonlipodystrophic patients (treated and untreated)
Hofman, Peter MacCallum Cancer Centre, Australia (2011) (Hofman and Hicks 2011)	1 (1)	40-year-old woman with squamous cell carcinoma of the cervix	Diffusely increased white adipose activity was attributed to insulin given prior to FDG administration. It was hypothesized that insulin administration caused hypoglycemia, resulting in activation of white adipose tissue to release fatty acids
Sharp, Cincinnati Children's Hospital Medical Center, USA (2012) (Sharp et al. 2012)	11 (unknown)	Children with lymphoblastic lymphoma (range: 2–15 years old)	Out of the 11 patients in this study, six were found to have increased white fat uptake, all of whom recently underwent induction chemotherapy which included high dose corticosteroids
Zade, Tata Memorial Hospital, India (2012) (Zade et al. 2012)	1 (0)	34-year-old man with HIV on HAART	The association of diffuse FDG activity in subcutaneous fat with lipodystrophy was noted, and the patient was switched from stavudine to tenofovir
Pattison, Peter MacCallum Cancer Centre, Australia (2014) (Pattison et al. 2014)	2 (0)	35-year-old man with non-small cell lung carcinoma and brain metastases as well as a 14-year-old boy with Hodgkin lymphoma	Both patients received high dose corticosteroids days before imaging with FDG-PET/CT. The lipolysis, lipogenesis, and adipogenesis needed to create a Cushingoid distribution of white adipose tissue were hypothesized to contribute to this pattern of uptake
Hwang, Catholic Kwandong University College of Medicine, Korea (2016) (Hwang et al. 2016)	1 (1)	61-year-old woman with diffuse large B-cell lymphoma	Abnormal FDG biodistribution was hypothesized to be due to the prednisone given during R-CHOP treatment or herbal treatments that contain corticosteroids
Kong, British Columbia Children's Hospital, Canada (2018) (Kong and Nadel 2018)	1 (1)	9-year-old girl with chronic Epstein-Barr virus infection	Chronic corticosteroid treatment was thought to be the cause of altered FDG biodistribution
Caton, Brigham and Women's Hospital, USA (2018) (Caton et al. 2018)	1 (0)	62-year-old man with HIV on HAART	Diffuse FDG localization to subcutaneous and visceral fat was found to be consistent with HIV-associated lipodystrophy
Wong, C. S. Mott Children’s Hospital, USA (2020) (Wong et al. 2020)	13 (8)	Children and young adults (range: 7–23 years old), most of whom were diagnosed with leukemia or lymphoma except one patient diagnosed with Ewing sarcoma and another patient diagnosed with neuroblastoma	All identified patients with altered biodistribution received induction treatment which included corticosteroids. Areas of active disease were obscured by white fat activity, and repeat imaging one week later resulted in relatively normalized biodistribution sufficient to detect previously hidden lesions
Staack, Mayo Clinic, Arizona, USA (2020) (Staack et al. 2020)	1 (1)	59-year-old woman with lymphoma	In addition to chemotherapy, her treatment regimen also included dexamethasone to prevent cerebral edema. Therefore, altered FDG biodistribution was ascribed to glucocorticoid-induced Cushing syndrome
Young, Yale New Haven Hospital, USA (2021) (Young et al. 2021)	1 (0)	13-year-old boy with T-cell lymphoblastic lymphoma	Although the patient received high dose corticosteroids with induction chemotherapy, the authors hypothesized that L-asparaginase, which was administered only three hours prior to FDG-PET/CT, may have been responsible for diffuse white adipose uptake
Kapoor, University of Kentucky Chandler Medical Center, USA (2021) (Kapoor et al. 2021)	1 (0)	7-year-old boy with T-cell lymphoblastic lymphoma	Iatrogenic Cushing syndrome resulting from dexamethasone included in induction chemotherapy was favored as the cause of FDG localization to white fat
Bansal, All India Institute of Medical Sciences, India (2021) (Bansal et al. 2021)	1 (1)	38-year-old woman with cervical cancer	While the patient was treated with chemoradiation, altered FDG biodistribution was found to be potentially caused by an herbal medication for herpes zoster which could contain corticosteroids
Singhal, All India Institute of Medical Sciences, India (2023) (Singhal et al. 2023)	1 (1)	12-year-old girl with lymphoma	Imaging with FDG-PET/CT was performed for end-of-treatment evaluation after four cycles of chemotherapy. Incidentally, the patient had been receiving prednisolone for nephrotic syndrome for two weeks prior to imaging, resulting in diffuse white fat activity

Although most commonly described in pediatric patients having recently undergone induction chemotherapy, this rare pattern of FDG uptake has been described in other patient populations as well

**Fig. 2:**
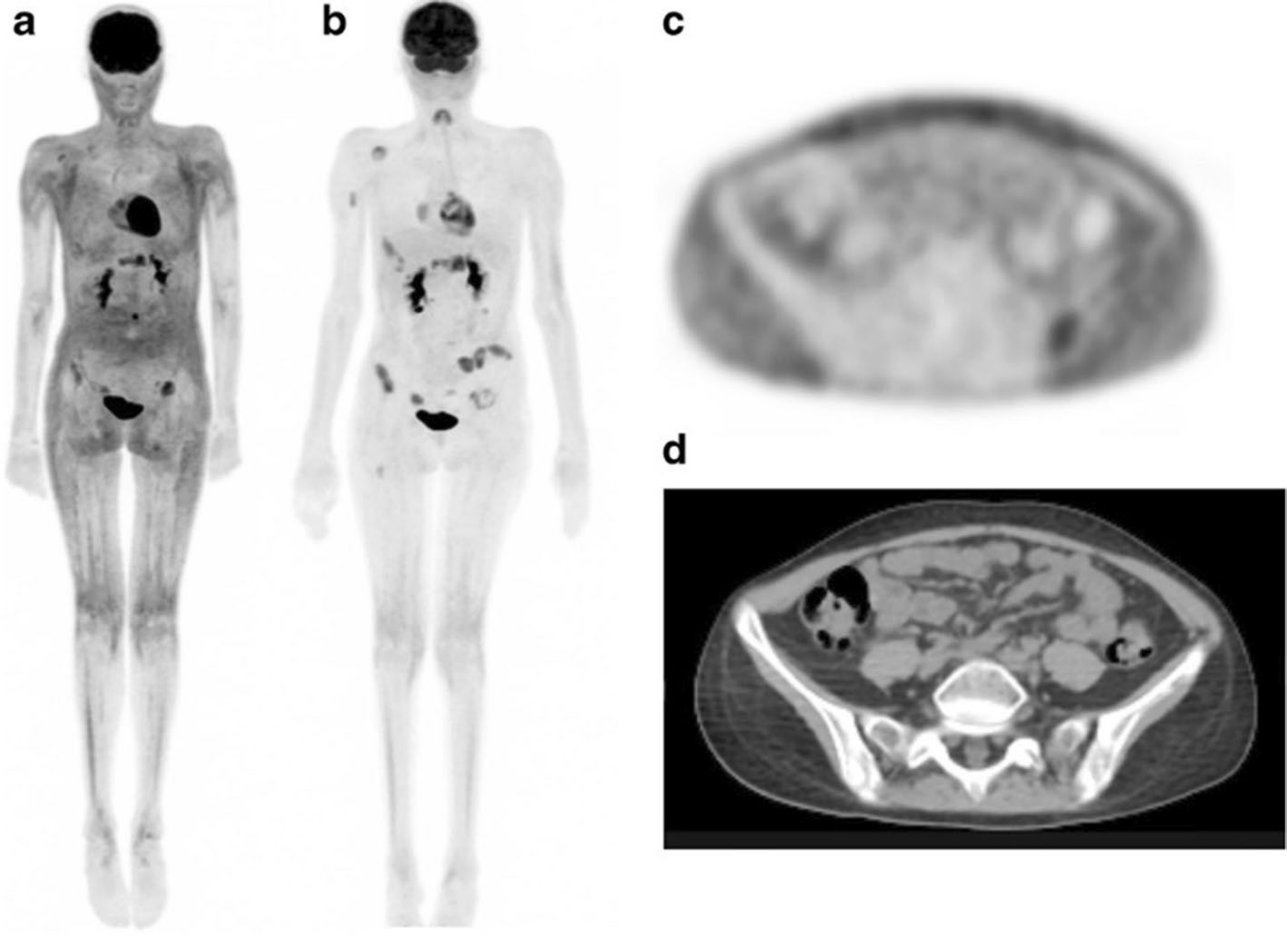
FDG-PET/CT maximum-intensity projection (MIP) in a 14-year-old patient as presented by Wong et al. with acute myeloid leukemia showing abnormal biodistribution of FDG localizing to subcutaneous white adipose tissue (**A**). SUVmax in the white fat of the flanks and gluteal regions was found to be 1.8 (**C** and **D**). Repeat FDG-PET/CT one week later (**B**) showed normalization of FDG biodistribution, revealing metabolically active osseous lesions in the proximal right humerus, right scapula, ribs and right femur, which were previously obscured. Image reprinted without changes from Wong et al. (2020)

**Fig. 3:**
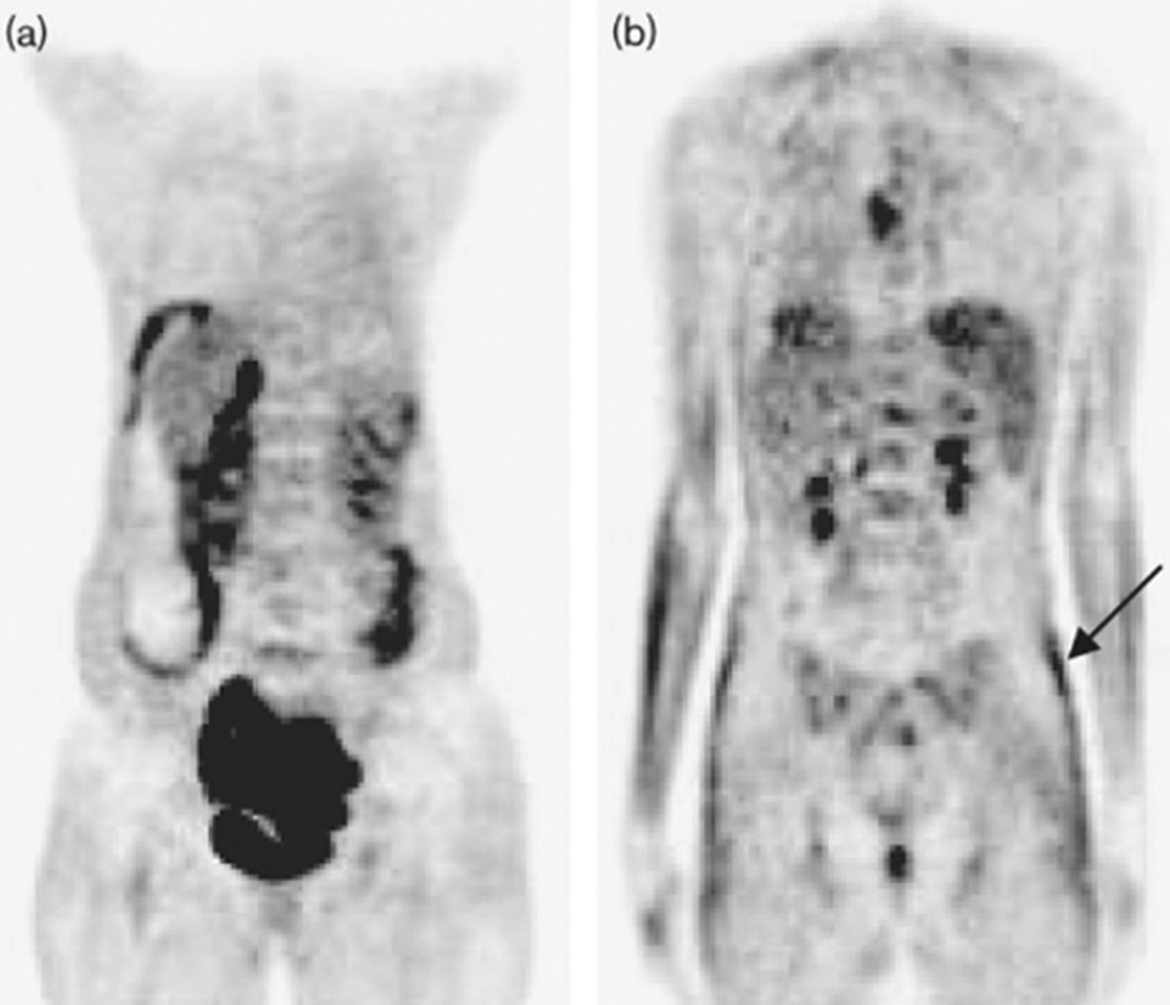
Coronal FDG-PET/CT images of two patients diagnosed with HIV infection and receiving highly active antiretroviral therapy (HAART). While low subcutaneous uptake of FDG is typically observed (**A**), certain patients with lipodystrophy are found to have increased white fat FDG uptake (**B**, arrow). Image reprinted without changes from Sathekge et al. (2010)

Regarding the potential effects of altered biodistribution on image interpretation, Wong et al. report a case in which osseous metastases were initially obscured by white adipose tissue activity in a pediatric lymphoma patient (Fig. 2) (Wong et al. 2020). The missed lesions were subsequently identified upon repeat imaging one week later, which had allowed for adequate normalization of FDG biodistribution. Four cases of adults with lymphoma (Bansal et al. 2021; Staack et al. 2020; Hwang et al. 2016; Pattison et al. 2014) as well as one case of a child with chronic active Epstein-Barr virus (Kong and Nadel 2018) have been reported with similar FDG-PET findings of increased white fat activity. Diffusely increased FDG localization to subcutaneous fat has also been described in patients diagnosed with human immunodeficiency virus (HIV) infection and experiencing lipodystrophy as a side effect of highly active antiretroviral therapy (HAART) (Fig. 3) (Sathekge et al. 2010).

In conclusion, we present a case and discuss the very rare entity of altered biodistribution of FDG, resulting in increased tracer uptake in subcutaneous white adipose tissue secondary to glucocorticoid use. This phenomenon that has only been reported in a very few preliminary studies, most commonly in pediatric leukemia and lymphoma patients receiving corticosteroids. In such cases, diagnostic accuracy is adversely affected, and repeat imaging at least one week after discontinuation of corticosteroids appears sufficient for overall normalization of FDG activity.

## Data Availability

Not applicable.

## Abbreviations

FDG: ^18^F-fluorodeoxyglucose
PET/CT: Positron emission tomography/computed tomography
DS: Deauville score
G-CSF: Granulocyte colony-stimulating factor
HIV: Human immunodeficiency virus
HAART: Highly active antiretroviral therapy

## References

[CR1] Angiolillo AL et al (2021) Excellent outcomes with reduced frequency of vincristine and dexamethasone pulses in standard-risk B-lymphoblastic leukemia: results from children’s oncology group AALL0932. J Clin Oncol 39(13):1437–1447 33411585 10.1200/JCO.20.00494 PMC8274746

[CR2] Bansal H et al (2021) Diffuse white adipose tissue 18F-FDG uptake-an unusual finding on 18F-FDG PET/CT. Clin Nucl Med 46(10):e513–e514 33867453 10.1097/RLU.0000000000003652

[CR3] Barrington SF et al (2014) Role of imaging in the staging and response assessment of lymphoma: Consensus of the International Conference on Malignant Lymphomas Imaging Working Group. J Clin Oncol 32(27):3048–3058 25113771 10.1200/JCO.2013.53.5229 PMC5015423

[CR4] Bleeker-Rovers CP et al (2004) F-18-fluorodeoxyglucose positron emission tomography for visualization of lipodystrophy in HIV-infected patients. AIDS 18(18):2430–2432 15622321

[CR5] Caton MT Jr, Miskin N, Hyun H (2018) 18F-FDG uptake in subcutaneous fat preceding clinical diagnosis of human immunodeficiency virus-associated lipodystrophy. Clin Nucl Med 43(12):e475–e476 30325831 10.1097/RLU.0000000000002298

[CR6] Cheson BD (2018) PET/CT in lymphoma: current overview and future directions. Semin Nucl Med 48(1):76–81 29195620 10.1053/j.semnuclmed.2017.09.007

[CR7] Gallamini A et al (2007) Early interim 2-[18F]fluoro-2-deoxy-D-glucose positron emission tomography is prognostically superior to international prognostic score in advanced-stage Hodgkin’s lymphoma: a report from a joint Italian-Danish study. J Clin Oncol 25(24):3746–3752 17646666 10.1200/JCO.2007.11.6525

[CR8] Haas B et al (2012) Targeting adipose tissue. Diabetol Metab Syndr 4(1):43 23102228 10.1186/1758-5996-4-43 PMC3568051

[CR9] Hanaoka K et al (2011) Fluorodeoxyglucose uptake in the bone marrow after granulocyte colony-stimulating factor administration in patients with non-Hodgkin’s lymphoma. Nucl Med Commun 32(8):678–683 21499162 10.1097/MNM.0b013e328346b32a

[CR10] Hofman MS, Hicks RJ (2011) White fat, factitious hyperglycemia, and the role of FDG PET to enhance understanding of adipocyte metabolism. EJNMMI Res 1(1):2 22214514 10.1186/2191-219X-1-2 PMC3192468

[CR11] Hwang DY et al (2016) Causes of (18)F-FDG uptake on white adipose tissue. Hell J Nucl Med 19(1):7–9 26929935 10.1967/s0024499100332

[CR12] Kapoor H et al (2021) Iatrogenic Cushing’s syndrome on (18)F-FDG-PET/CT: a pitfall in metabolic assessment of oncologic response. Clin Imaging 75:27–29 33486149 10.1016/j.clinimag.2021.01.010

[CR13] Kong MC, Nadel HR (2018) 18F-FDG PET/CT with diffusely high FDG uptake throughout subcutaneous adipose tissues. Clin Nucl Med 43(10):762–763 30036247 10.1097/RLU.0000000000002216

[CR14] Meignan M et al (2009) Report on the first international workshop on interim-PET-scan in lymphoma. Leuk Lymphoma 50(8):1257–1260 19544140 10.1080/10428190903040048

[CR15] Mikhaeel NG et al (2005) FDG-PET after two to three cycles of chemotherapy predicts progression-free and overall survival in high-grade non-Hodgkin lymphoma. Ann Oncol 16(9):1514–1523 15980161 10.1093/annonc/mdi272

[CR16] Nakatani K, Nakamoto Y, Togashi K (2012) Risk factors for extensive skeletal muscle uptake in oncologic FDG-PET/CT for patients undergoing a 4-h fast. Nucl Med Commun 33(6):648–655 22395030 10.1097/MNM.0b013e328352290f

[CR17] Oliveira AL et al (2015) Visceral and subcutaneous adipose tissue FDG uptake by PET/CT in metabolically healthy obese subjects. Obesity 23(2):286–289 25522219 10.1002/oby.20957 PMC4310760

[CR18] Pattison DA et al (2014) Enhanced white adipose tissue metabolism in iatrogenic Cushing’s syndrome with FDG PET/CT. J Clin Endocrinol Metab 99(9):3041–3042 24527716 10.1210/jc.2013-4090

[CR19] Sathekge M et al (2010) Evaluation of glucose uptake by skeletal muscle tissue and subcutaneous fat in HIV-infected patients with and without lipodystrophy using FDG-PET. Nucl Med Commun 31(4):311–314 20145581 10.1097/MNM.0b013e3283359058

[CR20] Sharp SE, Gelfand MJ, Absalon MJ (2012) Altered FDG uptake patterns in pediatric lymphoblastic lymphoma patients receiving induction chemotherapy that includes very high dose corticosteroids. Pediatr Radiol 42(3):331–336 21881935 10.1007/s00247-011-2228-7

[CR21] Singhal T et al (2023) Steroid-induced activated white adipose tissue detected on (18)F-FDG PET/CT. J Nucl Med Technol 51(2):158–159 37192824 10.2967/jnmt.122.265320

[CR22] Staack SO et al (2020) Glucocorticoid-induced hypermetabolism in white adipose tissue in Cushing syndrome. J Nucl Med Technol 48(3):285–286 31811068 10.2967/jnmt.119.237545

[CR23] Turcotte E et al (2006) Optimization of whole-body positron emission tomography imaging by using delayed 2-deoxy-2-[F-18]fluoro-D: -glucose Injection following I.V. Insulin in diabetic patients. Mol Imaging Biol 8(6):348–354 17053859 10.1007/s11307-006-0064-1

[CR24] Van Heertum RL et al (2017) Lugano 2014 criteria for assessing FDG-PET/CT in lymphoma: an operational approach for clinical trials. Drug Des Devel Ther 11:1719–1728 28670108 10.2147/DDDT.S136988 PMC5479259

[CR25] Wong KK et al (2020) 18F-2-fluoro-2-deoxyglucose uptake in white adipose tissue on pediatric oncologic positron emission tomography (PET)/computed tomography (CT). Pediatr Radiol 50(4):524–533 31776602 10.1007/s00247-019-04574-3

[CR26] Young CR et al (2021) Altered FDG biodistribution in subcutaneous white fat on PET/CT following l-asparaginase chemotherapy. Clin Nucl Med 46(3):e179–e180 33086270 10.1097/RLU.0000000000003340

[CR27] Zade A et al (2012) Hypermetabolic subcutaneous fat in patients on highly active anti-retroviral therapy treatment: subtle finding with implications. Indian J Nucl Med 27(3):183–184 23919073 10.4103/0972-3919.112726 PMC3728741

